# Supplementary material for: Artificial Intelligence in Surgical Training for Kidney Cancer: A Systematic Review of the Literature
Source: Diagnostics (Basel). 2023 Sep 27;13(19):3070. doi: 10.3390/diagnostics13193070 (PMC10572445; doi:10.3390/diagnostics13193070)
Supplement: Supplementary file 1 [file diagnostics-13-03070-s001.zip › Suppl files/Supp. File S4.pdf]

Search (7th of August 2023)
(no date or language filters)

1- **PubMed**
(("robotic assisted partial nephrectomy" OR "RAPN" OR "partial nephrectomy" OR "radical nephrectomy" OR "nephroureterectomy" OR "kidney cancer" OR "Renal cancer") AND ("Annotation" OR "machine learning" OR "Deep learning" OR "natural language processing" OR "computer vision" OR "artificial neural network" OR "artificial intelligence" OR "CV" OR "NLP" OR "DL" OR "ANN" OR "ML" OR "AI")) AND ("training" OR "performance assessment" OR "performance evaluation" OR "virtual reality" OR "VR" OR "augmented reality" OR "AR" OR "simulation" OR "workflow")

84 articles

2- **SCOPUS**
TITLE-ABS-KEY (("robotic assisted partial nephrectomy" OR "RAPN" OR "partial nephrectomy" OR "radical nephrectomy" OR "nephroureterectomy" OR "kidney cancer" OR "Renal cancer") AND ("Annotation" OR "machine learning" OR "Deep learning" OR "natural language processing" OR "computer vision" OR "artificial neural network" OR "artificial intelligence" OR "CV" OR "NLP" OR "DL" OR "ANN" OR "ML" OR "AI")) AND ("training" OR "performance assessment" OR "performance evaluation" OR "virtual reality"OR "VR" OR "augmented reality" OR "AR" OR "simulation" OR "workflow")

383 articles
